# Editorial: Metabolic conditions of chronic inflammatory diseases

**DOI:** 10.3389/fimmu.2022.1123676

**Published:** 2022-12-29

**Authors:** Anja Saalbach, Manfred Kunz

**Affiliations:** Department of Dermatology, Venereology and Allergology, University of Leipzig Medical Center, Leipzig, Germany

**Keywords:** psoriasis, lipedema, plasma cells, cytokines, obesity

Recent progress in the understanding of the pathogenesis of chronic inflammatory diseases such as rheumatoid arthritis, Crohn’s disease, psoriasis, lupus erythematosus, and multiples sclerosis have pointed to metabolic mechanisms as major driving forces in these diseases.

Western diet and lifestyle contribute to the development of obesity and degenerative diseases known as civilization diseases. Obesity is characterized by hyperglycemia, dyslipidemia, hyperinsulinemia, and an increase of adipose tissue associated with enhanced secretion of adipokines, chemokines, and cytokines. Overall, these metabolic disturbances are part of the so-called metabolic syndrome. Inflammatory cytokines may then impact on immune cell homeostasis and function, promoting a number chronic inflammatory diseases. Evidence has been provided for a number of these diseases that dietary measures may help to improve metabolic syndrome and at the same time improve disease activity of diseases such as psoriasis, psoriatic arthritis, rheumatoid arthritis, lupus, inflammatory bowel disease, multiple sclerosis and depression. The goal of the present article collection was to update current knowledge about the role of metabolic mechanisms active in chronic inflammatory diseases.

In their review article in this Research Topic, Saalbach and Kunz open a further avenue in this direction describing the link between psoriatic inflammation and bone metabolism. There is indeed mounting evidence that psoriasis is pathogenetically linked to osteoporosis, a metabolic disease with high clinical impact. Here, the pro-inflammatory cytokine IL-17, a major mediator in psoriasis pathogenesis, appears to play a central role. Recent reports of this group and others showed that osteocalcin and procollagen type 1 N-terminal propeptide, both serum markers for bone formation, show reduced expression in this disease. These recent findings merit further in depth analyses in future clinical trials linking bone metabolism to chronic inflammation.

In their original article in this issue, Schielke et al. describe the role of the chemokine CXCL16 in psoriasis-associated metabolic syndrome with regard to its impact on innate lymphoid cells. CXCL16 is a secreted and membrane bound chemokine that exerts pro-inflammatory activities, but is also known as a scavenger receptor for oxidized low density lipoproteins. Authors show enhanced expression of CXCL16 in monocyte subsets in psoriasis patients, and this expression correlated with psoriasis severity, body mass index and the risk to develop cardiovascular disease. Innate lymphoid cells, which are present in enhanced numbers in psoriasis lesions show high expression of CXCL16 receptor CXCR6 in obese patients. Moreover, CXCR6 expression was only found in tissue homing receptor (CCR7-) negative innate lymphoid cells, which led to the conclusion that these cells may migrate to CXCL16 high expressing, monocytic cells present in the skin. Overall, this work presented a nice link between obesity, innate immune cells and tissue inflammation in psoriasis.

In their original article, Wolf et al. show a link between inflammatory macrophage subtypes and lipedema. Lipedema is a chronic adipose tissue disease with a painful and irregular increase of the subcutaneous fat tissue of the extremities. Authors analyzed lipedema biopsies by Cytometry by Time-of-Flight and RNA sequencing to characterize the macrophage composition and signaling pathways involved in the chronic inflammation in this disease. Patients with lipedema showed a significant increase in the number of immunosuppressive (M2-type) macrophage populations in lipedema lesions.

A detailed enrichment analysis of the transcriptomic profiles of the myeloid compartment showed gene patters of activated MAP kinase signaling and PIK3 signaling. Both pathways are essential drivers of the polarization of macrophages toward the M2 phenotype. *In vitro*, IPI-549, a selective PI3Kγ inhibitor which switched M2 macrophages towards an M1-like phenotype resulted in a downregulation of M2 macrophage marker CD163. Finally, conditioned medium of macrophages was used to differentiate adipose-derived stem cells. The addition of IPI-549 resulted in a significant reduction of the lipid accumulation in adipose-derived stem cells. Furthermore, the extracellular metabolic potential of differentiated adipose-derived stem cells was normalized, further supporting a role of M2 macrophages in adipose tissue inflammation.

In their minireview in this Research Topic, Foh et al. discuss the impact of nutrients and metabolites on plasma cells, the major producers of immunoglobulins involved in autoimmunity, and also targets of recent anti-inflammatory antibody therapy. Emerging evidence suggests a key role of nutrients and microbial metabolites in the regulation and differentiation of plasma cells. They describe a number of pre-clinical models where metabolic parameters impacted on plasma cell function. For example, immunoglobulin class switching in a murine system was dramatically decreased under glucose restriction *in vitro*. Moreover, at low doses, short chain fatty acids propionate and butyrate enhanced class-switching. The short chain fatty acid butyrate can further enhance anti-inflammatory IL-10 producing plasma cells. In murine models of autoimmune diseases, it is also described that caloric restriction reduced the development of anti-nuclear antibody production, based on experiments in genetically modified disease-prone mice. The link between diet and plasma cell activity is further supported by the fact that ablation of B cells in high-fat diet-fed mice effectively abrogated the development of insulin resistance. Overall, these findings may link B cell activity to the gut microbiota, since the levels of different short chain fatty acids depend on specific bacterial strains within the gut microbiota.

Taken together, the mentioned studies supported a role of metabolic processes in chronic inflammatory diseases and also provided a link to antibody production in autoimmune diseases.

## Author contributions

All authors listed have made a substantial, direct, and intellectual contribution to the work and approved it for publication.

